# Adapentpronitrile, a New Dipeptidyl Peptidase-IV Inhibitor, Ameliorates Diabetic Neuronal Injury Through Inhibiting Mitochondria-Related Oxidative Stress and Apoptosis

**DOI:** 10.3389/fncel.2018.00214

**Published:** 2018-07-18

**Authors:** Lu Yang, Wenli Han, Ying Luo, Xiangnan Hu, Ying Xu, Huan Li, Congli Hu, Dan Huang, Jie Ma, Yang Yang, Qi Chen, Yuke Li, Jiahua Zhang, Hui Xia, Zhihao Chen, Hong Wang, Dongzhi Ran, Junqing Yang

**Affiliations:** ^1^Department of Pharmacology, The Key Laboratory of Biochemistry and Molecular Pharmacology, Chongqing Medical University, Chongqing, China; ^2^Laboratory Animal Center, Chongqing Medical University, Chongqing, China; ^3^Department of Pharmacology, The Laboratory of Pharmaceutical Chemistry, Chongqing Medical University, Chongqing, China; ^4^Department of Pharmaceutical Sciences, School of Pharmacy and Pharmaceutical Sciences, University at Buffalo, The State University of New York (SUNY), Buffalo, NY, United States; ^5^Department of Pharmacology, The Laboratory of Pharmaceutical Analysis, Chongqing Medical University, Chongqing, China

**Keywords:** adapentpronitrile, DPP-IV inhibitor, neuron injury, mitochondrial apoptosis pathway, reactive oxygen species

## Abstract

Our previous studies indicated that adapentpronitrile, a new adamantane-based dipeptidyl peptidase-IV (DPP-IV) inhibitor, has a hypoglycemic effect and ameliorates rat pancreatic β cell dysfunction in type 2 diabetes mellitus through inhibiting DPP-IV activity. However, the effect of adapentpronitrile on the neurodegenerative diseases has not been studied. In the present study, we first found that adapentpronitrile significantly ameliorated neuronal injury and decreased amyloid precursor protein (APP) and amyloid beta (Aβ) expression in the hippocampus and cortex in the high fat diet/STZ rat model of diabetes. Furthermore, adapentpronitrile significantly attenuated oxidative stress, downregulated expression of the pro-apoptotic proteins BAX, cytochrome c, caspase-9, and caspase-3, and upregulated expression of the anti-apoptotic protein Bcl-2, although there was no effect on GLP-1R expression. At 30 min post-injection of adapentpronitrile (50 mg/kg) *via* the tail vein, its concentration in normal rat brain was 0.2034 ± 0.0094 μg/g. Subsequently, we further confirmed the neuroprotective effects and mechanism of adapentpronitrile in HT22 cells treated with high glucose (HG) and aluminum maltolate [Al(mal)_3_] overload, respectively. Our results showed significant decreases in mitochondrial membrane potential (MTP) and Bcl-2 expression, accompanied by a significant increase in apoptosis, reactive oxygen species (ROS) generation, and the expression of pro-apoptotic proteins in HT22 cells exposed to these stimuli. Adapentpronitrile treatment protected against neuronal injury, suppressed ROS generation, and reduced MTP and mitochondrial apoptosis in HT22 cells; however, DPP-IV activity was not detected. Our results suggest that adapentpronitrile protects against diabetic neuronal injury, at least partially, by inhibiting mitochondrial oxidative stress and the apoptotic pathway in a DPP-IV-independent manner.

## Introduction

Diabetes is a multifactorial metabolic disease characterized by hyperglycemia and high morbidity. The incidence of diabetes has increased sharply in the last decade. Cognitive impairment induced by neuronal injury is one of the chronic complications of diabetes (Maher and Schubert, 2009; Biessels and Reagan, 2015; Baglietto-Vargas et al., 2016). Epidemiological studies show that diabetes significantly increases the risk of dementia and may finally develop into Alzheimer’s disease. β-Amyloid peptide (Aβ), a neuropathological hallmark of AD, is accumulated in specific brain regions (Hardy and Selkoe, 2002). Recent research demonstrates that insulin increases extracellular Aβ levels by elevating its secretion from neurons or modulating γ-secretase activity, and decreasing Aβ degradation by inhibiting Aβ-degrading enzymes (Gasparini et al., 2002; Phiel et al., 2003; Qiu and Folstein, 2006; Dineley et al., 2014). In addition, numerous studies have demonstrated that oxidative stress and mitochondrial abnormalities are common to the etiologies of both diabetes and Alzheimer’s disease (Bosco et al., 2011; Moreira, 2012). Hence, anti-diabetic agents might have important clinical and social value in the treatment of AD.

Mitochondria are essential organelles with multiple functions in energy metabolism, reactive oxygen species (ROS) generation, and apoptosis induction. Neurodegenerative diseases, which are induced by inflammation, oxidative stress, and metal and glucose toxicity, are usually accompanied by mitochondrial dysfunctions (Lin and Beal, 2006; Kumar and Gill, 2009; Verdile et al., 2015; Kandimalla et al., 2016; Koliaki and Roden, 2016). Furthermore, abnormal Aβ and tau proteins were shown to have a direct impact on mitochondrial function (Schmitt et al., 2012). Amyloid precursor proteins (APP) accumulates in mitochondrial import channels, thereby resulting in mitochondrial dysfunction (Devi et al., 2006). DuBoff et al. (2012) revealed that mitochondrial elongation was significantly increased in hippocampal neurons from tau transgenic mice. Thus, mitochondrial dysfunction is implicated as a critical step toward neuronal death.

Although ROS play a vital role in cellular survival and signaling pathways at physiological condition (cellular cycle regulation, phagocytosis, and enzyme activation), excessive ROS lead to a series of harmful effects including DNA, lipid and protein damage, mitochondrial dysfunction, or even cell death (Knock and Ward, 2011; Verbon et al., 2012; Son et al., 2013). Because mitochondria are both targets and sources of ROS, oxidant-induced mitochondrial dysfunction may lead to an increased production of superoxide anion radicals by the electron transport chain, thereby triggering a “vicious cycle” (Wallace, 2005). Oxidative stress is an important causative factor in the pathogenesis of aging, diabetes, and neurodegenerative diseases, which also cause remarkable accumulation of Aβ (Smith et al., 2007; Valko et al., 2007; Chen et al., 2012; Manoharan et al., 2016). In fact, persistent mitochondrial dysfunction and oxidative stress contribute to apoptosis *via* the mitochondria-dependent caspase cascade induced by the release of cytochrome c into the cytosol (Wallace, 2005).

The incretin hormone glucagon-like-peptide 1 (GLP-1), secreted by enteroendocrine L-cells in response to ingestion of nutrients, plays an important role in stimulating insulin secretion, ameliorating glycemic control, and repairing β-cell function. Since GLP-1 is degraded rapidly by dipeptidyl peptidase-IV (DPP-IV), the inhibitors of which have been regarded as appropriate agents to maintain blood glucose levels. Pipatpiboon et al. (2013) found that DPP-IV inhibitor vildagliptin could increase GLP-1 levels in both plasma and brain, restore neuronal insulin receptor function, and prevent brain mitochondrial dysfunction, thus ameliorating cognitive function caused by high-fat diet (HFD) consumption. Saxagliptin ameliorates Aβ, tau phosphorylation, and inflammatory markers in a streptozotocin-induced model of Alzheimer’s disease by increasing GLP-1 levels in the hippocampus (Kosaraju et al., 2013). Saxagliptin is also regarded as a novel therapeutic target for Parkinson’s disease *via* antioxidant, anti-inflammatory, and antiapoptotic mechanisms (Nassar et al., 2015). However, the lack of evidence demonstrating the ability of these DPP-IV inhibitors to penetrate the blood–brain barrier (Golightly et al., 2012), and the role of DPP-IV inhibitors in the neuroprotective mechanisms remain to be clarified.

Our previous study showed that adapentpronitrile (APPN, CMD-05), an adamantane-based anti-diabetic agent synthesized in our laboratory, exerted DPP-IV inhibitory activity *in vitro*, and also mediated hypoglycemic functions in diabetic rats (Ma et al., 2017). Considering the neuroprotective effects of vildagliptin and saxagliptin, we hypothesized that the new DPP-IV inhibitor adapentpronitrile represents a novel agent for the protection against neurodegenerative disease and neuronal injury. The aim of the present study was to investigate the neuroprotective effect and mechanisms of adapentpronitrile in diabetic rat from the flowing aspects: (1) the effects of adapentpronitrile on the neuronal injury were observed in diabetic rat induced by HFD/STZ and in HT22 cells induced by HG/aluminum maltolate [Al(mal)_3_]. (2) To primarily explore the mechanism of adapentpronitrile in neuronal injury. (3) Whether the neuroprotective mechanism of adapentpronitrile was related to the classic DPP-IV-dependent pathway, the adapentpronitrile concentration, and GLP-1R expression in rat brain and the DPP-IV activity in HT22 cells were detected.

## Materials and Methods

### Chemicals

Adapentpronitrile (CMD-05; 98.9% purity) was synthesized by Laboratory of pharmaceutical chemistry, Chongqing Medical University (Patent apply number: 201610818878.5), and its purity was detected by high-performance liquid chromatography (HPLC).

### Animals and Protocol

Sprague-Dawley (SD) male rats were obtained from Animal Laboratory Administrative Center, Chongqing Medical University (Chongqing, China), and housed in the barrier housing facility (SPF scale), which according with national standard “Laboratory Animal-Requirements of Environment and Housing Facilities.” The care and experimental operation of animal have conforming to “Chongqing Administration Rule of Laboratory Animal.” The experimental procedures were approved by the animal laboratory administrative center and the institutional ethics committee of Chongqing Medical University (License number: SYXK YU 2012-0001) and also in accordance with the National Institutes of Health guidelines. All experiments reviewed and approved by the Instructional Animal Care and Use Committee (IACUC).

**FIGURE 1:**
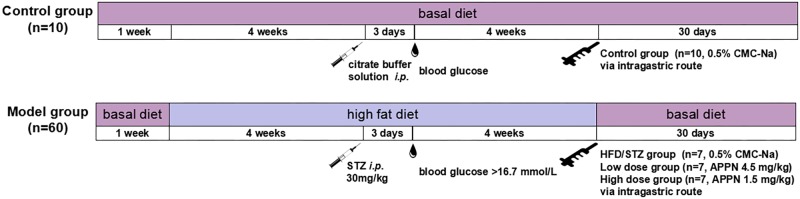
The general procedure of this study *in vivo*.

The experiments were performed in 70 male rats (aged 9 weeks, 80–100 g). The rats were housed in controlled conditions of temperature (22 ± 2°C), relative humidity (50 ± 10%), and 12/12-h light/dark cycle with water *ad libitum*. After a week of acclimation, 10 rats were randomly selected and fed with basal diet as the control group, 60 rats were fed a HFD (20% sugar, 10% lard, 10% egg yolk, and 60% basal feed) for 4 weeks to induce insulin resistance (Ma et al., 2017). Diabetes was induced by a single intraperitoneal injection of streptozotocin (Solarbio, China, S8050; STZ, 30 mg/kg), which is particularly toxic to pancreatic beta cells in mammals. Control rats were injected with the same volume of vehicle (citrate buffer solution). Hyperglycemia was defined as blood glucose >16.7 mmol/L, 72 h after STZ injection. Diabetic rats were maintained on the HFD for 4 weeks and randomly divided into following groups (*n* = 7): HFD/STZ group (0.5% CMC-Na), low dose group (adapentpronitrile 1.5 mg/kg), high dose group (adapentpronitrile 4.5 mg/kg). The dose of adapentpronitrile was based on our previous study (Ma et al., 2017). Diabetic rats in the adapentpronitrile groups were chronically administered adapentpronitrile (4.5 or 1.5 mg/kg) for 30 days *via* the intragastric route, while the control and model groups received the same amount of vehicle (0.5% CMC-Na; **Figure 1**).

After administration, three rats in each group were randomly selected and used for histopathological examination, and the remaining four rats were used for biochemical examination and Western blot.

### HE Staining Observation

Three rats in each group were randomly selected and transcardially perfused with phosphate-buffered saline (PBS). Then, the brain tissues were isolated and fixed overnight in 4% paraformaldehyde. After embedded by paraffin, serial sections (5 μm) of brain tissue were obtained, which were stained by hematoxylin–eosin (HE). Proceed as follows: dewaxing in xylen, hydration in alcohols, HE staining, dehydration in alcohols, transparent in xylen and mounting with neutral resins. Ten consecutive fields were selected randomly from the hippocampal and cortical neurons, and the morphological changes were observed under an optical microscope (Olympus, Japan) at 400× magnification (Guo et al., 2016).

### Measurement of SOD Activity and MDA Content

After anesthesia of another four rats in each group, the cortex and hippocampus were separated on an ice plate and washed by normal saline. Then the isolated tissues were dried and stored at −80°C until use. The superoxide dismutase (SOD) activity and malondialdehyde (MDA) content were measured using the Total Superoxide Dismutase Assay Kit (Beyotime, China, S0101) and the Lipid Peroxidation MDA Assay Kit (Beyotime, China, S0131), respectively. The protein content was determined using the BCA Protein Assay Kit (Beyotime, China, P0010S).

### Measurement of Adapentpronitrile Concentration

Male SD rats (200–220 g) were injected a single dose of adapentpronitrile (50 mg/kg, *n* = 4) *via* tail vein after fasting 12 h. At 30 min post-injection, blood samples were collected from abdominal aorta and the brain tissues were isolated on ice (Ma et al., 2017). Plasma was obtained after centrifugation. Then the plasma and isolated brain tissues were stored at −80°C until used.

Pipette 100-μL plasma samples and 200-μL acetonitrile [containing 500-ng internal standard (IS)] into 1.5-ml eppendorf tube. After that, the mixture was centrifuged for supernatant at 12,000 × *g* for 15 min at 4°C.

The brain tissues were weighed and homogenized in a twofold volume of acetonitrile containing 500 ng. Briefly, acetonitrile was used to homogenize brain tissue according to a ratio of 2-mL acetonitrile to 1-g tissue sample. The homogenates were centrifuged for supernatant at 12,000 × *g* for 15 min at 4°C.

Adapentpronitrile concentration in plasma and brain were determined using an HPLC system equipped with a UV detector. An octadecyl endcapped Phecda-C_18_ column (250 mm × 4.5 mm, 5-μm particle size) and Waters universal injector (100 μL capacity) were used. The optimum mobile phase was identified and consisted of acetonitrile and 10 mmol/L ammonium acetate (40:60, vol/vol). Samples (50 μL) were injected and a flow rate of 1 mL/min was equilibrated. The elution was monitored at 204 nm. The system was operated at the ambient temperature. Calibration curve was constructed by plotting standard peak area vs. concentration. Recoveries were calculated as the ratio of peak-area of the analyte from the fortified samples to the corresponding peak-area ratio of standard solutions.

### Cell Culture

Immortalized murine hippocampal HT22 cell lines were obtained from BNCC, China. HT22 cells were cultured in DME/F12 medium (Hyclone, United States) supplemented with 10% fetal bovine serum (FBS; Hyclone, United States) and 1% 100× penicillin–streptomycin (Gibco, United States) at 37°C and 5% CO_2_.

### *In Vitro* Glucose (HG)/Al(mal)_3_ Overload Model

HT22 cells were plated in 96-well plates (8 × 10^4^ neurons/mL). The cells were divided into the control group (mannitol 100 mM) and four HG overloaded groups (glucose 25–100 mM). The cells were divided into the control group (maltol 600 μM) and four aluminum overloaded groups [Al(mal)_3_ 50–400 μM; Ma et al., 2016]. After incubation, the relevant indicators were detected and the optimum concentrations of HG and Al(mal)_3_ were selected in following experiments.

HT22 cells were divided into the control, HG overload and adapentpronitrile intervention groups induced by HG. HT22 cells were divided into the control group, Al(mal)_3_ overloaded groups and adapentpronitrile intervention groups induced by Al(mal)_3_. After incubation, the relevant indicators were detected in following experiments.

### MTT Assay

HT22 cells were cultured in the 96-well plates at 8 × 10^4^ cells/mL and subjected to HG or Al(mal)_3_ as described above. After the intervention, 3-(4,5-dimethyl-thiazol-2-yl)-2,5-diphenyl-tetrazolium bromide (MTT, 20 μL, 5 mg/ml; Sigma, United States, M2128) was added per well. After 4 h of incubation, the medium was removed, and 150 μL dimethyl sulfoxide (DMSO) was added to solubilize the purple formazan. Then, the plate shook slowly on the horizontal shaking table free from light for 10 min at room temperature. Finally, optical density (OD) was detected at 490 nm using a microplate reader (BioTek, United States; Lobner, 2000).

### LDH Leakage Rate Detection

Cell death was evaluated using the lactate dehydrogenase (LDH) Cytotoxicity Assay Kit (Beyotime, China, C0017). HT22 cells were cultured in 96-well plates at 5 × 10^4^ cells/ml. After HG or Al(mal)_3_ treatment for 36 h, the LDH leakage rate in the cell culture supernatant was measured according to the manufacturer’s instruction.

### Flow Cytometry Analysis

HT22 cells were seeded in six-well plates at 8 × 10^4^ cells/mL and exposed to HG or Al(mal)_3_ as described above. After the incubation period, cells were trypsinized without EDTA, collected and suspended in 1 ml PBS. Apoptosis was determined by flow cytometry using the Annexin V-FITC/propidium iodide (annexin V/PI) apoptosis detection kits according to the manufacturer’s protocol. Three separate experiments were performed.

### Transmission Electron Microscopy Observation

The changes in mitochondrial ultrastructure were confirmed by transmission electron microscopy (TEM) examination. After treatment, HT22 cells were collected and fixed in glutaraldehyde solution at 4°C. The fixed samples were sent to the electron microscope center of Chongqing Medical University for microscopic observed and photographed.

### DPP-IV Activity Determination

Supernatant of HT22 cells and lysed HT22 cells were collected to measure DPP-IV activity in control group. Culture medium and equivoluminal lysis buffer were collected and regarded as blank group. DPP-IV enzyme assay was carried out according to the manufacturer’s instructions. DPP-IV Activity Assay Kits (AnaSpec, United States, AS-24098) were used according the manufacturer’s instructions. In this assay, DPP4 cleaves a substrate [H-Gly-Pro-7-amino-4-methyl coumarin (AMC)] to release a quenched fluorescent group, AMC, which can be easily detected using a fluorescence microplate reader at excitation and emission wavelengths of 354 and 442 nm, respectively.

### TUNEL Assays

HT22 cells were cultured in cover slides and washed in ice-cold PBS. Samples were fixed with 4% paraformaldehyde for 15 min and permeabilized in PBS containing proteinase K for 3 min at room temperature. Apoptosis was determined using TUNEL FITC Apoptosis Detection Kits (Vazyme Biotech, China, A111-02) according to the manufacturer’s protocol. Apoptotic cells were observed using a fluorescence microscopy. Four consecutive fields were selected randomly and the apoptosis rate was calculated as the ratio of the number of apoptotic cells to the total number of cells (expressed as a percentage).

### Mitochondrial ROS Assay

Mitochondrial ROS levels were measured using ROS Assay Kits (Beyotime, China, S0033). 2′,7′-Dichloro-dihydro-fluorescein diacetate (DCFH-DA) is a non-fluorescent probe that can be oxidized to the highly fluorescent derivative DCF by intracellular ROS. The cells were stained with 2-μM DCFH-DA and incubated at 37°C for 20 min. Four consecutive fields were selected randomly and the fluorescence intensity was measured under a fluorescence microscope after removal of the culture medium.

### Mitochondrial Membrane Potential (Δψ_m_) Assay

The mitochondrial membrane potential (MMP, Δψ_m_) was measured using Mitochondrial Membrane Potential Assay Kits with JC-1 (Beyotime, China, C2006). JC-1, a cationic fluorescent dye, can be accumulated in the mitochondrial matrix to form polymer, which gives off a strong red fluorescence. At low MMP, JC-1 exists in the form of monomer in the cytoplasm and yields green fluorescence. The cells were strained with JC-1 dye at 37°C for 20 min. After washing with dilution buffer, four consecutive fields were selected randomly and the fluorescence intensity was measured under a fluorescence microscope.

### Western Blot Analysis

After treatment, HT22 cells were washed in ice-cold PBS and lysed by RIPA lysis buffer containing phosphatase and protease inhibitors. The samples were collected and centrifuged at 12,000 × *g* and 4°C for 15 min, after incubated for 20 min on ice. The supernatant was collected and the total protein concentrations were measured with a BCA protein assay kit (Beyotime, China, P0010S). Loading buffer (Beyotime, China, P0015L) was added into the remaining supernatant and boiled at 100°C for 10 min. Ultimately, samples were stored at −20°C for further research.

Forty milligrams of rat cortex or hippocampus (*n* = 4) was added to 0.4 ml of RIPA Lysis Buffer for protein extraction and was centrifuged at 12,000 × *g* for 15 min at 4°C after homogenization. The supernatant was collected and determined with the BCA protein assay kit (Beyotime, China, P0010S) for protein concentrations. The remaining supernatant was mixed with Loading buffer (Beyotime, China, P0015L) and boiled at 100°C for 10 min. Finally, samples were stored at −20°C for further research.

According to the Cell Mitochondrial Isolation kit (Beyotime, China, C3601), the mitochondria can be separated quickly and conveniently, and the cytoplasmic protein can be obtained to study the release of mitochondrial proteins to the cytoplasm, such as cytochrome c.

Equal amounts of protein (20 mg) were separated by sodium dodecyl sulfate polyacrylamide gel electrophoresis (SDS–PAGE). The proteins were then transferred to PVDF membranes (Millipore, United States) and blocked with 5% bovine serum albumin (BSA) in Tris-buffered saline/Tween-20 (TBST) buffer for 4 h at room temperature. Membranes were probed overnight at 4°C with primary antibodies for specific detection of APP (dilution 1:500; Boster, China, BA0581), Aβ (dilution 1:1000; Abcam, United Kingdom, ab62658), GLP-1R (dilution 1:500, Bioss, China, bs-1559R), Bcl-2 (dilution 1:500; Abcam, United Kingdom, ab196495), Bax (dilution 1:400; Proteintech, China, 50599-2-Ig), cytochrome c (dilution 1:1000; Abcam, United Kingdom, ab133504), caspase-9 (dilution 1:1000; Abcam, United Kingdom, ab184786), caspase-3 (dilution 1:1000; Abcam, United Kingdom, ab184787), and β-actin (dilution 1:3000; Proteintech, United States, 60008-1-Ig; **Table 1**). The blots were incubated with HRP-conjugated secondary antibodies (dilution 1:2000; **Table 1**) at room temperature for 1 h. Finally, immunoreactive bands were visualized by ECL (Bio-Rad, United States) and quantified using Image Lab software (Bio-Rad, United States). All experiments were performed in triplicate.

**Table 1:** List of primary and secondary antibodies used in the study.

	Host	Dilution	Supply	Cat. No.	Application
Primary antibody					
APP	Rabbit	1:500	Boster	BA0581	WB
Aβ	Rabbit	1:1000	Abcam	ab62658	WB
GLP-1R	Rabbit	1:500	Bioss	bs-1559R	WB
Bcl-2	Rabbit	1:500	Abcam	ab196495	WB
Bax	Rabbit	1:400	Proteintech	50599-2-Ig	WB
Cytochrome c	Rabbit	1:1000	Abcam	ab133504	WB
Caspase-9	Rabbit	1:1000	Abcam	ab184786	WB
Caspase-3	Rabbit	1:1000	Abcam	ab184787	WB
β-Actin	Mouse	1:3000	Proteintech	60008-1-Ig	WB
Secondary antibody					
Anti-mouse HRP	Goat	1:2000	Proteintech	SA00001-1	WB
Anti-rabbit HRP	Goat	1:2000	Proteintech	SA00001-2	WB

### Statistical Analysis

All investigators were complete randomization in the implementation process of experiments. All experiments were repeated at least four times, and representative results are shown. Data were presented as mean ± SD. Statistical analysis was carried out using SPSS 17.0 (SPSS Inc. Chicago, IL, United States). Normal distribution of data and homogeneity of variance were determined by Shapiro–Wilk test and one-way analysis of variance (ANOVA) test, respectively, followed by Dunnett-t type multiple comparison tests. *P* < 0.05 was considered to indicate statistical significance.

## Results

### Effects of Adapentpronitrile on Pathomorphology and the Expression of APP and Aβ Proteins in Hippocampus and Cortex of Rat

**FIGURE 2:**
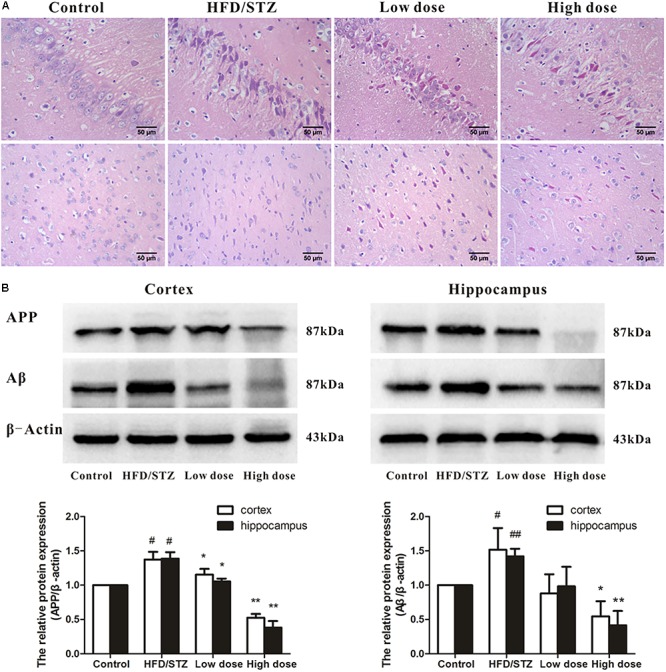
Effects of adapentpronitrile on pathomorphology and the expression of APP and Aβ proteins in hippocampus and cortex of rat. **(A)** Representative neuronal pathomorphology in hippocampus and cortex. Sections were pictured at 400×. Scale bar = 50 μm. **(B)** The expressions of APP and Aβ proteins were measured by Western Blotting. Data were expressed as mean ± SD of four independent experiments, and were analyzed statistically using one-way ANOVA, followed by Dunnett-t type multiple comparison tests. ^#^*P* < 0.05 and ^##^*P* < 0.01 vs. Control group, respectively; ^∗^*P* < 0.05 and ^∗∗^*P* < 0.01 vs. HFD/STZ group, respectively.

Histopathological examination revealed that the neurons were in clear and intact structure, and arranged regularly in the control group. By contrast, HFD/STZ group showed severe hippocampal and cortical neurons injuries including karyopyknosis, hyperchromatic nuclei, and cell loss. Administration of adapentpronitrile (4.5 mg/kg) obviously ameliorated the pathomorphological injury in the hippocampus and cortex (**Figure 2A**).

As shown in **Figure 2B**, the expression of APP and Aβ proteins elevated significantly in HFD/STZ-treated rat, while adapentpronitrile (4.5 mg/kg) significantly blunted the changes of APP and Aβ protein expressions.

### Effects of Adapentpronitrile on Oxidative Stress in the Hippocampus and Cortex of Rat

**FIGURE 3:**
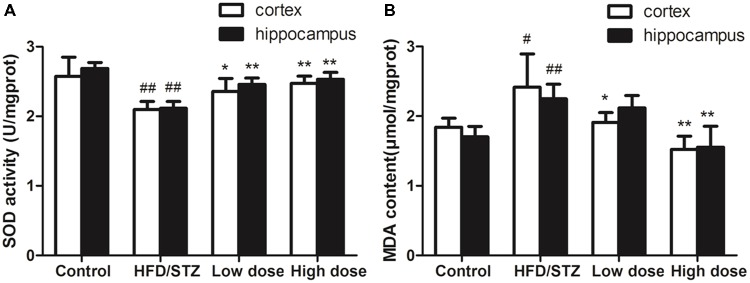
Effects of adapentpronitrile on oxidative stress in the hippocampus and cortex of rat. **(A)** The change of SOD activity in hippocampus and cortex. **(B)** The alteration of MDA content in hippocampus and cortex. Data were expressed as mean ± SD of four independent experiments, and were analyzed statistically using one-way ANOVA, followed by Dunnett-t type multiple comparison tests. ^#^*P* < 0.05 and ^##^*P* < 0.01 vs. control group, ^∗^*P* < 0.05 and ^∗∗^*P* < 0.01 vs. HFD/STZ group, respectively.

Both hippocampal and cortex SOD activity in HFD/STZ group significantly decreased compared with the control group. Adapentpronitrile treatment reversed the decrease of SOD activity (**Figure 3A**). Both hippocampal and cortex MDA content in HFD/STZ group significantly increased compared with the control group. Adapentpronitrile administration significantly blunted the increase of MDA content in HFD/STZ rats (**Figure 3B**).

### Effects of Adapentpronitrile on GLP-1R and Mitochondria-Dependent Apoptosis Protein Expression Induced by HFD/STZ in the Rat Hippocampus and Cortex

**FIGURE 4:**
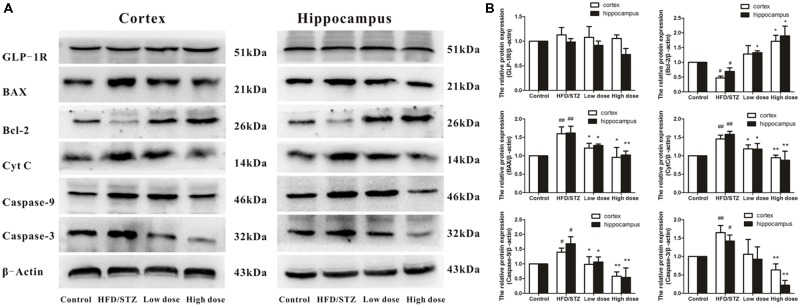
Effects of adapentpronitrile on GLP-1R and mitochondria-dependent apoptosis protein expression induced by HFD/STZ in the rat hippocampus and cortex. **(A)** The expressions of GLP-1R and mitochondria-dependent apoptosis proteins were measured by Western Blotting. Representative images of experiments are shown. **(B)** The relative expression of GLP-1R and mitochondria-dependent apoptosis proteins were standardized to endogenous β-actin protein for each sample. Data were expressed as mean ± SD of four independent experiments, and were analyzed statistically using one-way ANOVA, followed by Dunnett-t type multiple comparison tests. ^#^*P* < 0.05 and ^##^*P* < 0.01 vs. Control group, respectively; ^∗^*P* < 0.05 and ^∗∗^*P* < 0.01 vs. HFD/STZ group, respectively.

Glucagon-like-peptide 1 is an incretin hormone, which plays a role in controlling synaptic plasticity and reversing memory impairment. As shown in **Figure 4**, there were no significant difference among these four groups in the protein expressions of GLP-1R. On the basis of this evidence, we deduced that the mechanism underlying the protective effects of adapentpronitrile may be GLP-1 independent.

Therefore, we also examined the impact of adapentpronitrile on the expression of the apoptosis-related proteins. The expression of the anti-apoptotic Bcl-2 was significantly decreased in HFD/STZ group, whereas the expressions of the pro-apoptotic proteins Bax, cytochrome c, caspase-9, and caspase-3 were significantly increased. However, all the alterations were significantly reversed by adapentpronitrile (4.5 mg/kg) treatment (**Figure 4**).

### Concentration of Adapentpronitrile in the Rat Plasma and Brain Tissue

**FIGURE 5:**
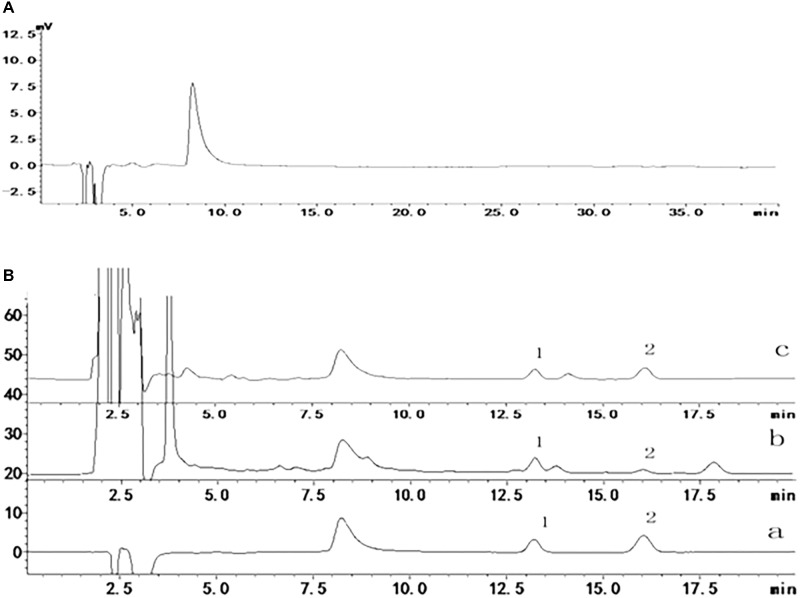
The specificity of adapentpronitrile detected by HPLC-MS. **(A)** Acetonitrile **(B)** Sample (1) carbamazepine (2) adapentpronitrile (a) standard sample, (b) plasma, (c) brain.

To confirm the ability of adapentpronitrile to permeate the BBB to protective against neuronal apoptosis, we used a HPLC method to determine the concentration of adapentpronitrile in rat plasma and brain tissue. According to HPLC methodology, the maximum ultraviolet absorption of adapentpronitrile is 204 nm, the quantitation limit and the detection limit were 0.03 μg/ml (S/N = 10) and 0.09 μg/ml (S/N = 3), respectively. The standard curve of adapentpronitrile had good linear relation in the range of 0.1–10 μg/mL in plasma, the equation was *Y* = 0.5267*X*–0.0796 (*r* = 0.9997, *n* = 4). The intra-day and inter-day precision RSD were 3.66–8.71 and 4.33–5.59%, respectively. The recovery of adapentpronitrile was 82.19 ± 6.57–118.21 ± 3.72%, and which has good stability in 48 h. The linear relation of adapentpronitrile was excellent within the range of 0.09–5 μg/ml in brain tissue; the equation was *Y* = 0.325*X*–0.0125 (*r* = 0.9999, *n* = 4). The intra-day and inter-day precision RSD were 1.71–5.69 and 3.77–4.54%, respectively. The recovery of adapentpronitrile was 106.68 ± 2.47–117.50 ± 5.10 and which has good stability in 48 h. These methods are stable, sensitive, and practicable for examination of adapentpronitrile distribution. As shown in **Figure 5**, HPLC-MS was confirmed to have good specificity to detect adapentpronitrile.

As shown in **Table 2**, the adapentpronitrile concentrations in plasma and brain were 2.8002 ± 0.5691 μg/100 μL and 0.2034 ± 0.0094 μg/g, respectively. Therefore, these data confirmed that adapentpronitrile permeates the BBB.

**Table 2:** Concentration of APPN in rat plasma and brain (mean ± SD, *n* = 4).

Sample	Unit	Concentration
Plasma	μg/ml	28.002 ± 5.691
Brain	μg/g	0.2034 ± 0.0094

### Establishment of HG Overload Model in HT22 Cells

**FIGURE 6:**
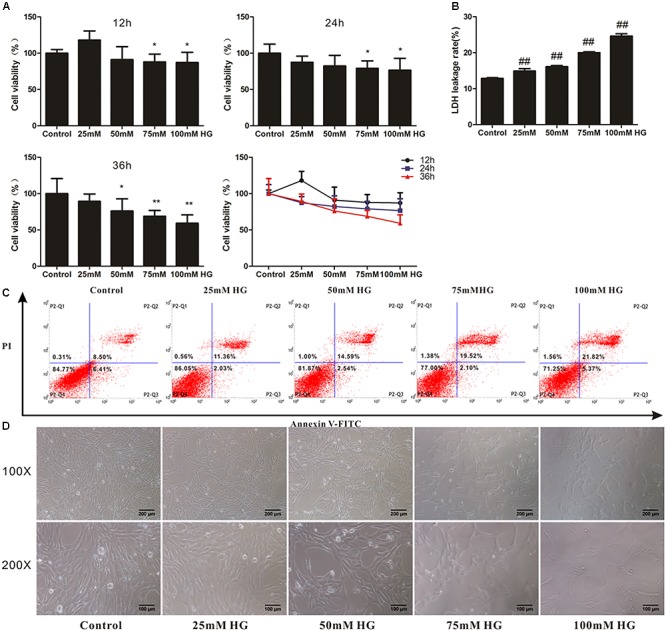
The effects of HG overload on HT22 cells. **(A)** The viability of HT22 cells treated with different concentrations of glucose. Data were expressed as mean ± SD of six independent experiments, and were analyzed statistically using one-way ANOVA, followed by Dunnett-t type multiple comparison tests. ^∗^*P* < 0.05, ^∗∗^*P* < 0.01 vs. the control group. **(B)** The LDH leakage rate of HT22 cells treated with different concentration of glucose. Data were expressed as mean ± SD of six independent experiments, and were analyzed statistically using one-way ANOVA, followed by Dunnett-t type multiple comparison tests. ^##^*P* < 0.01 vs. the control group. **(C)** The flow cytometry apoptosis analysis of HT22 cells were treated with high glucose for 36 h. The representative images of flow cytometry analysis are shown. **(D)** The cytomorphology changes in HT22 cells treated with different concentration of glucose. Representative images of experiments are shown. The magnification of images were 100× and 200×, respectively. Scale bars were 200 and 100 μm, respectively.

To determine whether adapentpronitrile could protect neurons from HG injury, HT22 cells were treated with appropriate concentration of glucose (HG) to establish the neuronal damage model *in vitro*. As is shown in **Figure 6A**, treatment of glucose caused cytotoxicity in HT22 cells in a time- and concentration-dependent manner, showing that treatment of 200-μM glucose for 36 h resulted in a suitable neuronal injury model (66.24%).

The LDH leakage rate and apoptosis rate were elevated with the increase of glucose concentration, and treatment of 75-mM glucose led to a suitable LDH leakage rate (20.02%) and apoptosis rate (21.62%) for model establishment (**Figures 6B,C**).

As shown in **Figure 6D**, the cell number decreased with concentration of glucose from 25 to 100 mM, illustrating that cell proliferation was inhibited by HG.

Based on these results, the treatment of 75 mM glucose for 36 h was used in further experiments.

### Adapentpronitrile Prevented HT22 Cells Against HG-Induced Cytotoxicity

**FIGURE 7:**
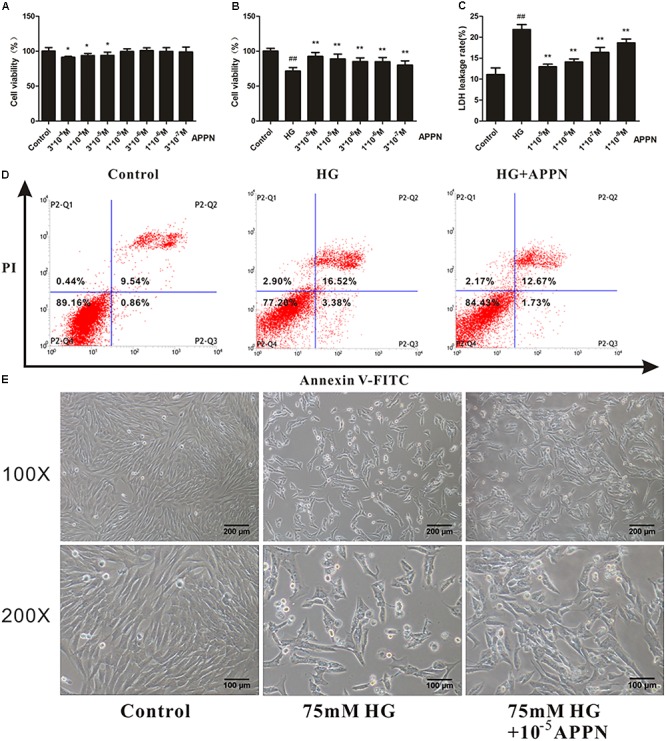
The effect of adapentpronitrile on HG-induced cytotoxicity in HT22 cells. **(A)** The cytotoxicity of adapentpronitrile on HT22 cells. Data were expressed as mean ± SD of six independent experiments, and were analyzed statistically using one-way ANOVA, followed by Dunnett-t type multiple comparison tests. ^∗^*P* < 0.05 vs. the control group. **(B)** The effect of adapentpronitrile on the viability in HT22 cells induced by HG-overload. **(C)** The effect of adapentpronitrile on the LDH leakage rate in HT22 cells induced by HG-overload. Data were expressed as mean ± SD of six independent experiments, and were analyzed statistically using one-way ANOVA, followed by Dunnett-t type multiple comparison tests. ^##^*P* < 0.01 vs. the control group; ^∗^*P* < 0.05 and ^∗∗^*P* < 0.01 vs. the HG group, respectively. **(D)** The effect of adapentpronitrile on apoptosis in HT22 cells induced by HG-overload. The representative images of flow cytometry analysis are shown. **(E)** The effect of adapentpronitrile on the changes of cytomorphology in HT22 cells induced by HG overload. Representative images of experiments are shown. The magnification of images were 100× and 200×, respectively. Scale bars were 200 and 100 μm, respectively.

Prior to explore the effect of adapentpronitrile on HG-induced cytotoxicity, the safety of adapentpronitrile on HT22 cells was evaluated by MTT assay. Treatment with adapentpronitrile at concentrations lower than 1 × 10^−5^ M did not caused significant cytotoxicity in HT22 cells (**Figure 7A**).

The results showed that treatment with adapentpronitrile significantly increased the viability and reduced LDH leakage rate induced by HG overload in a concentration-dependent manner (**Figures 7B,C**). Treatment with adapentpronitrile (1 × 10^−5^ M) could ameliorate the apoptosis rate and pathomorphological change induced by 75-mM HG (**Figures 7D,E**). Therefore, these results together indicated that adapentpronitrile can inhibit neuronal apoptosis induced by HG overload *in vitro*.

### DPP-IV Activity in HT22 Cells

**FIGURE 8:**
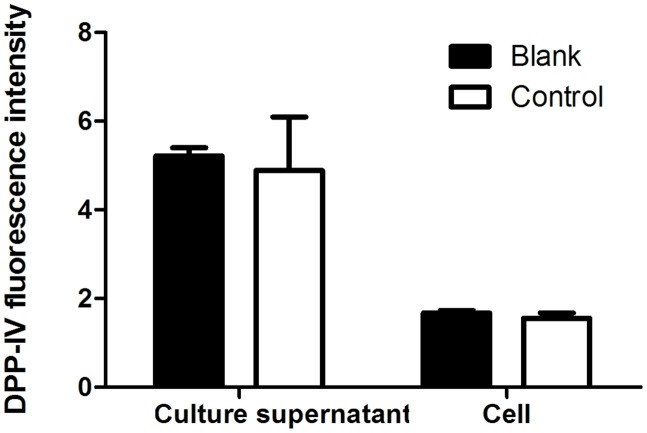
The DPP-IV activity in HT22 cells. Data were expressed as mean ± SD of six independent experiments, and were analyzed statistically using one-way ANOVA, followed by Dunnett-t type multiple comparison tests.

To confirm whether dipeptidyl peptidase IV (DPP-IV) exists in HT22 cells. Supernatant from both HT22 cells incubation and the HT22 cells lysate were collected to measure DPP-IV activity as the control group. Equivoluminal culture medium and lysis buffer were collected and regarded as the blank group. As shown in **Figure 8**, there was no significant difference in the fluorescence intensity between the culture supernatant of HT22 cells and that of the cell-free control. There was also no significant difference in the fluorescence intensity between HT22 cell lysates and that of lysis buffer. Therefore, we concluded that DPP-IV activity was not present in HT22 cells, and indicating the protective effect of adapentpronitrile on neuronal injury *in vitro* is independent of DPP-IV.

### Adapentpronitrile Protected Against HG-Induced Mitochondrial Apoptosis

**FIGURE 9:**
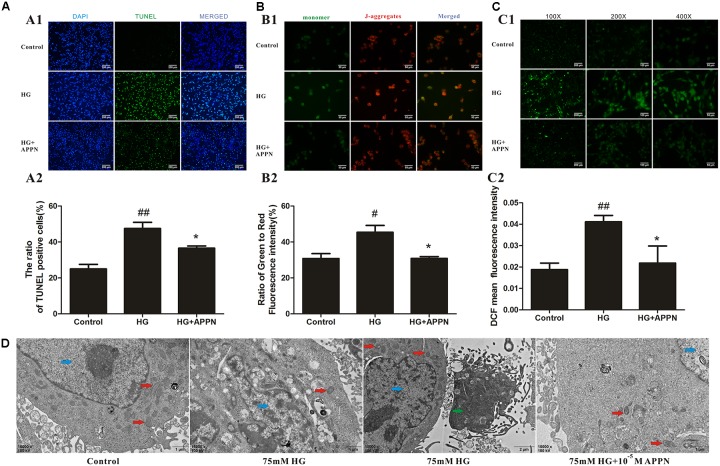
Effects of adapentpronitrile on Δψm, ROS level, apoptosis, and mitochondria morphology in HT22 cells induced by HG overload. **(A)** Effects of adapentpronitrile on the TUNEL-positive cell number in HT22 cells induced by HG overload. **(B)** Effects of adapentpronitrile on mitochondrial membrane potential in HT22 cells induced by HG overload. **(C)** Effects of adapentpronitrile on ROS generation in HT22 cells induced by HG overload. Representative images of experiments are shown. Data were expressed as mean ± SD of four independent experiments, and were analyzed statistically using one-way ANOVA, followed by Dunnett-t type multiple comparison tests. ^#^*P* < 0.05 and ^##^*P* < 0.01 vs. the control group, respectively; ^∗^*P* < 0.05 vs. the HG group. **(D)** The ultrastructure in HT22 cells detected by TEM. Representative images of experiments are shown. Sections were pictured at 15,000×. Scale bar = 1 μm. The blue, red, and green arrows were pointed to the nucleus, mitochondria, and apoptotic body, respectively.

As shown in **Figures 9A–C**, the numbers of TUNEL-positive cells and the ratio of green to red fluorescence of JC-1 and ROS level were significantly increased in the HG-overload group, while a reduction was noted in the adapentpronitrile-treated group.

The changes in mitochondrial ultrastructure were confirmed by TEM examination. As is shown in **Figure 9D**, in the control group, the nuclei of HT22 cells were round or oval with regular shape and evenly distributed chromatin, and the mitochondrial morphology was normal with a complete structure. In HG overload group, the chromatin was aggregated at the nuclear membrane, the mitochondrial cristae were thick and short accompanied with apoptotic bodies, thereby indicating the occurrence of mitochondrial fission; all alterations were ameliorated by adapentpronitrile treatment.

### Effect of Adapentpronitrile on Mitochondria-Dependent Apoptosis-Related Protein Expression Caused by HG in HT22 Cells

**FIGURE 10:**
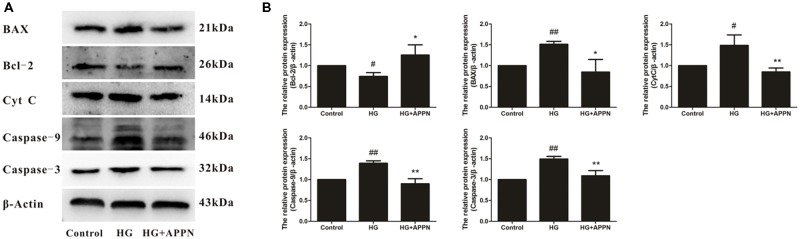
Effect of adapentpronitrile on mitochondria-dependent apoptosis-related protein expression caused by HG in HT22 cells. **(A)** Representative blots of adapentpronitrile on the expressions of apoptosis-related proteins in HT22 cells induced by HG-overload. **(B)** The relative of protein expression were standardized to endogenous β-actin protein for each sample. Data were expressed as mean ± SD of four independent experiments, and were analyzed statistically using one-way ANOVA, followed by Dunnett-t type multiple comparison tests. ^#^*P* < 0.05 and ^##^*P* < 0.01 vs. the control group; ^∗^*P* < 0.05, ^∗∗^*P* < 0.01 vs. the HG group.

As shown in **Figure 10**, exposure of HT22 cells to 75 mM HG for 36 h significantly decreased the expression of the anti-apoptotic protein Bcl-2 and increased the expression of the pro-apoptotic proteins Bax, cytochrome c, caspase-9, and caspase-3. All alterations in the expression of apoptosis-related proteins were reversed by adapentpronitrile treatment. Our results indicated that adapentpronitrile protects against HG-induced cell apoptosis *via* the mitochondrial apoptotic pathway.

### Establishment of Al(mal)_3_ Overload Model in HT22 Cells

**FIGURE 11:**
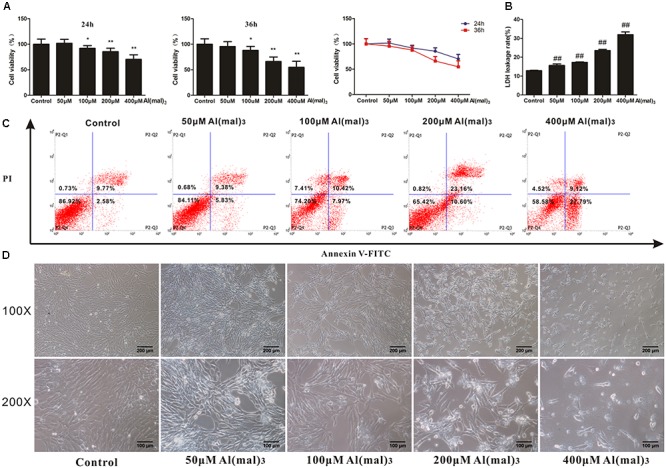
The effects of Al(mal)_3_ on HT22 cells. **(A)** The viability of HT22 cells treated with different concentrations of Al(mal)_3_. Data were expressed as mean ± SD of 10 independent experiments, and were analyzed statistically using one-way ANOVA, followed by Dunnett-t type multiple comparison tests. ^∗^*P* < 0.05, ^∗∗^*P* < 0.01 vs. the control group. **(B)** The LDH leakage rate of HT22 cells treated with different concentration of Al(mal)_3_. Data were expressed as mean ± SD of six independent experiments, and were analyzed statistically using one-way ANOVA, followed by Dunnett-t type multiple comparison tests. ^##^*P* < 0.01 vs. the control group. **(C)** The flow cytometry apoptosis analysis of HT22 cells were treated with Al(mal)_3_ for 36 h. The representative images of flow cytometry analysis are shown. **(D)** The cytomorphology changes of HT22 cells treated with different concentration of Al(mal)_3_. Representative images of experiments are shown. The magnification of images were 100× and 200×, respectively. Scale bars were 200 and 100 μm, respectively.

To determine whether adapentpronitrile could protect neurons from non-HG injury, HT22 cells were treated with appropriate concentration of Al(mal)_3_ to establish the neuronal damage model *in vitro*. As is shown in **Figure 11A**, treatment of Al(mal)_3_ caused cytotoxicity in HT22 cells in a time- and concentration-dependent manner, showing that treatment of 200 μM Al(mal)_3_ for 36 h resulted in a suitable neuronal injury model (66.24%).

The LDH leakage rate and apoptosis rates were elevated with the increase of Al(mal)_3_ concentration, and treatment of 200 μM Al(mal)_3_ led to a suitable LDH leakage rate (23.54%) and apoptosis rate (33.76%) for model establishment (**Figures 11B,C**).

As shown in **Figure 11D**, the cell number decreased with concentration of Al(mal)_3_ from 50 to 400 μM, while only 400 μM Al(mal)_3_ could contribute to a morphological change, illustrating that cell proliferation was inhibited by Al(mal)_3_.

Based on these results, the treatment of 200 μM Al(mal)_3_ for 36 h was used in further experiments.

### Adapentpronitrile Prevented HT22 Cells Against Al(mal)_3_-Induced Cytotoxicity

**FIGURE 12:**
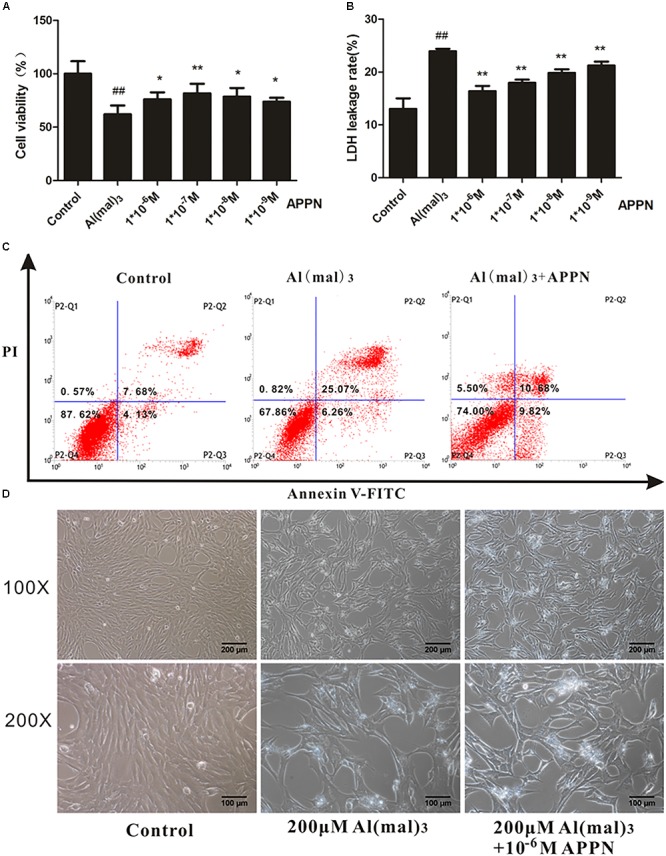
The effect of adapentpronitrile on Al(mal)3-induced cytotoxicity in HT22 cells. **(A)** The effect of adapentpronitrile on the viability in HT22 cells induced by Al(mal)_3_ overload. **(B)** The effect of adapentpronitrile on the LDH leakage rate in HT22 cells induced by Al(mal)_3_ overload. Data were expressed as mean ± SD of six independent experiments, and were analyzed statistically using one-way ANOVA, followed by Dunnett-t type multiple comparison tests. ^##^*P* < 0.01 vs. the control group; ^∗^*P* < 0.05 and ^∗∗^*P* < 0.01 vs. the Al(mal)_3_ group, respectively. **(C)** The effect of adapentpronitrile on apoptosis in HT22 cells induced by Al(mal)_3_-overload. The representative images of flow cytometry analysis are shown. **(D)** The effect of adapentpronitrile on the changes of cytomorphology in HT22 cells induced by Al(mal)_3_-overload. Representative images of experiments are shown. The magnification of images were 100× and 200×, respectively. Scale bars were 200 and 100 μm, respectively.

The results showed that treatment with adapentpronitrile significantly increased the viability and reduced LDH leakage rate induced by Al(mal)_3_-overload in a concentration-dependent manner (**Figures 12A,B**). Treatment with adapentpronitrile (1 × 10^−6^ M) could ameliorate the apoptosis rate and pathomorphological change induced by 200 μM Al(mal)_3_ (**Figures 12C,D**). Therefore, these results together indicated that adapentpronitrile can inhibit neuronal apoptosis induced by Al(mal)_3_ overload *in vitro*.

### Adapentpronitrile Protected Against Al(mal)_3_-Induced Mitochondrial Apoptosis

**FIGURE 13:**
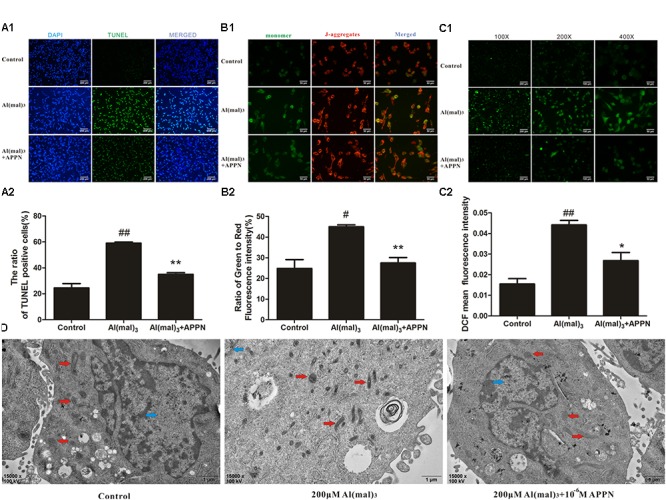
Effects of adapentpronitrile on Δψm, ROS level, apoptosis, and mitochondria morphology in HT22 cells induced by Al(mal)_3_ overload. **(A)** Effects of adapentpronitrile on the TUNEL-positive cell number in HT22 cells induced by Al(mal)_3_ overload. **(B)** Effects of adapentpronitrile on mitochondrial membrane potential in HT22 cells induced by Al(mal)_3_ overload. **(C)** Effects of adapentpronitrile on ROS generation in HT22 cells induced by Al(mal)_3_ overload. Representative images of experiments are shown. Data were expressed as mean ± SD of four independent experiments, and were analyzed statistically using one-way ANOVA, followed by Dunnett-t type multiple comparison tests. ^#^*P* < 0.05 and ^##^*P* < 0.01 vs. the control group, respectively; ^∗^*P* < 0.05 and ^∗∗^*P* < 0.01 vs. the Al(mal)_3_ group, respectively. **(D)** The ultrastructure in HT22 cells detected by TEM. Representative images of experiments are shown. Sections were pictured at 15,000×. Scale bar = 1 μm. The blue, red, and green arrows were pointed to the nucleus, mitochondria, and apoptotic body, respectively.

As shown in **Figures 13A–C**, the numbers of TUNEL-positive cells, the ratio of green to red fluorescence of JC-1 and ROS level were significantly increased in the Al(mal)_3_ overload group, while a reduction was noted in the adapentpronitrile-treated group. The photograph of TEM showed that the chromatin was aggregated at the nuclear membrane and the cristae of mitochondria was thick and short in the Al(mal)_3_-overloaded group, which indicated that mitochondrial fission was broadly raised, while all alterations were ameliorated by adapentpronitrile treatment (**Figure 13D**).

### Effect of Adapentpronitrile on Mitochondria-Dependent Apoptosis-Related Protein Expression Caused by Al(mal)_3_ in HT22 Cells

**FIGURE 14:**
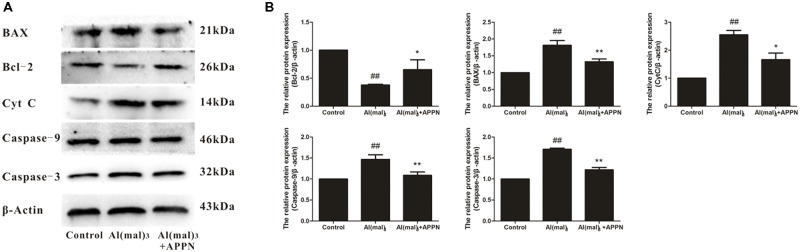
Effect of adapentpronitrile on mitochondria-dependent apoptosis-related protein expression caused by Al(mal)_3_ in HT22 cells. **(A)** Representative blots of adapentpronitrile on the expressions of apoptosis-related proteins in HT22 cells induced by Al(mal)_3_. **(B)** The relative of protein expression were standardized to endogenous β-actin protein for each sample. Data were expressed as mean ± SD of four independent experiments, and were analyzed statistically using one-way ANOVA, followed by Dunnett-t type multiple comparison tests. ^#^*P* < 0.05 and ^##^*P* < 0.01 vs. the control group; ^∗^*P* < 0.05, ^∗∗^*P* < 0.01 vs. the Al(mal)_3_ group.

As shown in **Figure 14**, the expression of Bcl-2 protein was significantly decreased in the Al(mal)_3_-overload group, whereas the expressions of Bax, cytochrome c, caspase-9, and caspase-3 protein were significantly increased. All the alterations of apoptosis-related proteins were reversed by adapentpronitrile treatment.

## Discussion

Alzheimer’s disease is an irreversible progressive neurodegenerative disorder characterized by cognitive deficits and memory loss. Despite various strategies have been explored, unfortunately, promising preclinical outcomes were generally disappointing (Mullard, 2016). In recent years, epidemiological studies suggest that diabetes mellitus is a risk factor for Alzheimer’s disease, which may include three common pathological properties as follows, cerebrovascular inflammation, amyloid deposition, and impairment of brain insulin signaling (Takeda et al., 2010; De Felice and Ferreira, 2014). Antidiabetic agents, therefore, may have value in the treatment of AD.

In the present study, neuronal injury in rats was induced by a HFD combined with low-dose STZ (30 mg/kg). It is postulated that a persistent high-fat and high-sucrose diet contributes to an accumulation of glucolipotoxicity and β cell dysfunction, thereby leading to insulin resistance and hyperinsulinemia (Li et al., 2015). STZ is particularly toxic to islet cells in mammals and has been utilized to establish T2DM model, which accelerates β cell dysfunction and insulin deficiency (Lenzen, 2008). The pathology of HFD/STZ model reflects the progression from insulin resistance to hyperglycemia and hypoinsulinemia associated with diabetes (Reed et al., 2000; Li et al., 2015). In our previous study, we observed a reduction in β cell numbers by approximately 40% in HFD/STZ rats, which consistent with those observed in reports of patients with diabetes in the clinic (Butler et al., 2003; Ma et al., 2017). It is well known that hippocampus and cortex play an important role in the learning, cognition, and memory, and is also one of the earliest signs of neurodegenerative disorder such as Alzheimer’s disease. Numerous investigations indicated that the atrophy and apoptosis of brain may occur in type 2 diabetes, especially in the hippocampus and cortex, and that may contribute to cognitive impairment (den Heijer et al., 2003; McCrimmon and Ryan, 2012; Ho and Sommers, 2013; Moran et al., 2013; Hirabayashi et al., 2016). In the present study, we observed significant pathomorphological changes and neuronal injury in the cortex and vulnerable hippocampal CA4 region following HFD/STZ insult. The protein expressions of APP and Aβ, which are a major hallmark in the pathologic progression of AD, were elevated significantly in both the hippocampus and cortex of the HFD/STZ rats. These results confirmed that the diabetic AD rat model was established successfully.

Treatment with adapentpronitrile, a new DPP-IV inhibitor, resulted in a dose-dependent amelioration of the pathological changes in the hippocampal CA4 region of T2DM rats. Furthermore, the abnormal protein expressions of APP and Aβ were reversed. These findings demonstrate the protective effects of adapentpronitrile against neuronal injury.

Glucagon-like-peptide 1, which is an incretin hormone secreted mainly by intestinal L-cells, plays a vital role in motivating insulin secretion, suppressing glucagon release, thus ameliorating glycemic control (Holst, 2007; Ma et al., 2014). In addition to the expression in peripheral tissues, the GLP-1 receptor is expressed in the brain, particularly in the hippocampus (Dunphy et al., 1998). Accumulating evidence demonstrates that GLP-1 analogs play a role in controlling synaptic plasticity and reversing memory impairment (Gault and Hölscher, 2008; McClean and Hölscher, 2014). Moreover, GLP-1 acts as a neurotropic factor by promoting proliferation and inhibiting apoptosis. The half-life of endogenous GLP-1 in the circulation is approximately 2 min due to its rapid degradation by DPP-IV (Drucker and Nauck, 2006; Ranganath, 2008). Therefore, DPP-IV inhibitors have been utilized to improve glycemic control in the treatment of T2DM since the first agent sitagliptin was approved by FDA in 2006 (Drucker and Nauck, 2006; Verspohl, 2009). DPP-IV inhibitors adapentpronitrile have been shown to stimulate insulin biosynthesis and secretion, improve glycemic control, and decrease HbA1c in diabetic rats induced by HFD/STZ (Ma et al., 2017). In the present study, adapentpronitrile significantly attenuated the neuronal injury in the hippocampus and cortex of rats caused by HFD/STZ. However, there was no significant difference among these groups in the protein expressions of the GLP-1R, indicating that the neuroprotective effects of adapentpronitrile are, at least partially, independent of the DPP-IV pathway. Therefore, the neuroprotective mechanism of adapentpronitrile remains to be fully elucidated.

The neurotoxic mechanism of HFD/STZ might be attributed to the overproduction of ROS. SOD is an important antioxidant enzyme, which functions primarily as a free radical scavenger to protect against oxidative stress. MDA, a biomarker of lipid peroxidation, is generated *via* a series of enzyme reactions (Del Rio et al., 2005). In the present study, we found that the SOD activity was significantly decreased and the MDA content was significantly increased in the brain of T2DM rat. Adapentpronitrile treatment reversed the abnormal decrease in SOD activity and increased MDA levels. These results further confirmed the involvement of oxidative stress in the rat model of diabetic neuronal injury induced by HFD/STZ, whereas adapentpronitrile treatment improved the oxidative imbalance.

Additionally, previous studies confirmed that oxidative stress promotes mitochondrial dysfunction. The mitochondrial respiratory chain can be impaired by mitochondrial dysfunction to produce excess ROS and exacerbate oxidative stress even further, forming a “vicious circle” (Vučetić-Arsić et al., 2013). Mitochondrial dysfunction initiates apoptosis by activating caspase-dependent or caspase-independent pathways (Arun et al., 2016). This process is regulated by the proteins of Bcl-2 family, including Bcl-2, Bcl-xL, Bax, and Bak. After a conformational shift, the pro-apoptotic proteins Bax and Bak insert themselves into mitochondrial membranes, where pro-apoptotic factors cytochrome c is released into the cytosol through the mitochondrial permeability transition pore (MPTP) or other channels (Tait and Green, 2010). Elevated cytosolic levels of soluble cytochrome c can lead to the increased cell mortality associated with neurodegeneration (Perier et al., 2005). In our present study, expression of the anti-apoptotic protein Bcl-2 was decreased significantly in the cortex and hippocampus of HFD/STZ rats, while the expression of mitochondria-associated pro-apoptotic proteins were increased significantly; these alterations were reversed by adapentpronitrile treatment. Thus, the current study indicates that the neuroprotective effects of adapentpronitrile are mediated *via* the mitochondrial apoptosis pathway.

Numerous studies suggest that disruption of the BBB is associated with age, obesity, and diabetes, and could contribute to early cognitive impairment (Tucsek et al., 2014; Montagne et al., 2015; van de Haar et al., 2016; Xu et al., 2016). Extensive research has revealed candidate drugs that have a better effect on CNS diseases in preclinical studies, while the clinical application has little or no effect. The main reason is that a candidate drug has to be administered directly into the cerebrospinal fluid, where it can enter the brain to mediate the neuroprotective effect (Pardridge, 2011). Therefore, it is necessary to determine whether a candidate drug can penetrate the BBB for the treatment of CNS diseases. In the present study, adapentpronitrile was detected in the brain of normal rats at 30 min after intravenous injection, indicating that adapentpronitrile can penetrate BBB. Hence, further studies are required to determine whether the neuroprotective effects of adapentpronitrile are mediated direct or indirect action on neurons and whether the mechanism is related to DPP-IV dependent or independent.

To imitate the hyperglycemic state in the CNS of diabetic patients, we subjected HT22 cells, which are a neuronal cell line derived from murine hippocampus, to HG treatment *in vitro*. We found that neuronal injury aggravated with the HG concentration increasing, which was ameliorated by adapentpronitrile treatment.

In accordance with the results of our *in vitro* study, adapentpronitrile significantly abrogated the increase in ROS levels and neuronal apoptosis, and the decrease in MMP in HT22 cells following HG insult. Furthermore, adapentpronitrile treatment significantly increased Bcl-2 protein expression in HT22 cells, while the expression of Bax, cytochrome c, caspase 3, and caspase 9 proteins was decreased.

In order to further confirm the neuroprotection and mechanism of adapentpronitrile, the cell injury model was established by aluminum overload in HT22 cells. Metals play a risk role in AD *via* the production of free radicals, including iron, aluminum, mercury, copper, and zinc (Christen, 2000). Extensive literature illustrated that the neurotoxic effects of aluminum are beyond any doubt, and it cannot be discarded that aluminum may contribute to the development of AD (Gupta et al., 2005). Epidemiological evidence indicated that aluminum concentrations of drinking water is associated to the prevalence of AD with a dose–response relationship, and a similar link between exposure and the prevalence were also reported in elderly populations (Crapper et al., 1980; Rondeau et al., 2009). Furthermore, elevation of aluminum content were observed in the brains of patients with AD (Becaria et al., 2002), and it was also detected in *post mortem* neurofibrillary tangles and senile plaques of AD patients (Meiri et al., 1993). Both aluminum and aggregated β-amyloid stimulate free radicals production and contribute to mitochondrial dysfunction relating to the pathology of AD. Al(mal)_3_ is a salt forms of aluminum used to imitate AD-like neuronal injury *in vivo* and *in vitro* for its stability at physiological pH (Johnson et al., 2005). Our experimental results showed that treatment of Al(mal)_3_ decreased the viability and increase the LDH leakage rate in a concentration-dependent manner, which was ameliorated by adapentpronitrile treatment. Adapentpronitrile significantly blunted the reduction of mitochondrial membrane potential, ROS overproduction, of TUNEL-positive cell number and pro-apoptotic protein expressions triggered by Al(mal)_3_-overload.

To explore the association of the neuroprotective effect of adapentpronitrile with DPP-IV inhibition, we determined DPP-IV activity in HT22 cells. We did not detect significant activity of DPP-IV in HT22 cells, indicating that the neuroprotective effects of adapentpronitrile are mediated *via* a DPP-IV-independent pathway *in vitro*.

## Conclusion

The DPP-IV inhibitor adapentpronitrile effectively attenuates the neuronal injury caused by HG/Al(mal)_3_ overload *in vitro* and ameliorates both the hippocampal and cortical neuron injury caused by HFD combined with low dose STZ *in vivo*. Pharmacokinetic studies confirmed the ability of adapentpronitrile to penetrate the blood–brain barrier *in vivo*, while no significant DPP-IV activity was detected in HT22 cells *in vitro*. These results together suggest that adapentpronitrile mediates obvious protection against diabetic neuronal injury, at least partially, by inhibiting mitochondrial oxidative stress and the apoptosis pathway *via* a DPP-IV-independent pathway. Thus, adapentpronitrile is implicated as a promising candidate for AD therapy in the clinical setting. However, the mechanism underlying the neuroprotective effects of adapentpronitrile and the potential of other whether the other DPP-IV inhibitors to mediate similar effects remain to be fully explored.

## Author Contributions

JY made substantial contribution to conception and design and performance of the study. LY, WH, YiL, HL, CH, DH, JM, YY, QC, YuL, JZ, HX, ZC, HW, and, DR participated in performance of all experiments and carried out the data analysis. XH provided adapentpronitrile and experimental guidance. YX participated in the grammar and writing instruction. LY participated in performance of the study and in writing the manuscript. All authors read and approved the final manuscript. JY is the guarantor of this work and, as such, had full access to all the data in the study and takes responsibility for the integrity of the data and the accuracy of the data analysis.

## Conflict of Interest Statement

The authors declare that the research was conducted in the absence of any commercial or financial relationships that could be construed as a potential conflict of interest.
